# Synchronic development of hydrocele and groin hernia during pregnancy: a case report

**DOI:** 10.1186/s13256-023-04099-2

**Published:** 2023-09-27

**Authors:** Lise Rasmussen, Waqas Farooqui, Annedorte Ries, Morten Willer Stadeager

**Affiliations:** 1 Gastrounit, Copenhagen University Hospital – Amager and Hvidovre, 2650 Hvidovre, Denmark; 2 Department of Pathorlogy, Copenhagen University Hospital – Amager and Hvidovre, Hvidovre, Denmark

**Keywords:** Case report, Hernia, Hydrocele, Canal of Nuck

## Abstract

**Introduction:**

Hydroceles of the canal of Nuck are rare, and have not been described in relation to pregnancy.

**Case presentation:**

A 34-year-old Caucasian female patient had bilateral groin swelling debuted during her pregnancy. A preoperative magnetic resonance imaging scan found bilateral hydroceles of the canal of Nuck. Operative findings and histological examinations revealed a left-sided inguinal hernia and a right-sided hydrocele. The patient was discharged well and without signs of postoperative complications or hernia recurrence.

**Discussion:**

In this case, a hydrocele and a hernia sac were morphologically identical in terms of preoperative appearance and development. Given the morphological correlation, it was surprising to find different operative findings confirmed by the histopathological examination.

**Conclusion:**

This is the first ever report of the synchronic development of two morphologically identical cystic processes, with one being a hydrocele and the other a hernia sac. In addition, the hydrocele developed during pregnancy, making this case even more unique.

## Introduction

Hydroceles of the canal of Nuck are rare, and have not been described in relation to pregnancy [1]. Groin hernias during pregnancy are also rare, with a prevalence of 0.12% [2]. Groin hernias are not associated with any risks during pregnancy, and herniotomy can, if necessary, take place after delivery. In this case report, a synchronic development of an inguinal hernia and hydrocele is described. Furthermore, the onset of disease is correlated with pregnancy.

## Presentation of case

A 34-year-old Caucasian female patient, a former professional athlete, was referred to our outpatient clinic with intermittent swelling and localized discomfort in both groin regions over a period of 2 years. Symptoms exacerbated during strenuous exercise and heavy lifting, and impaired her daily routines.

Swelling debuted during her pregnancy, and remained unchanged after delivery. The pregnancy was a first time, single-fold, with natural delivery of a healthy child. The patient was healthy, age equivalent, and a non-smoker.

**Fig. 1:**
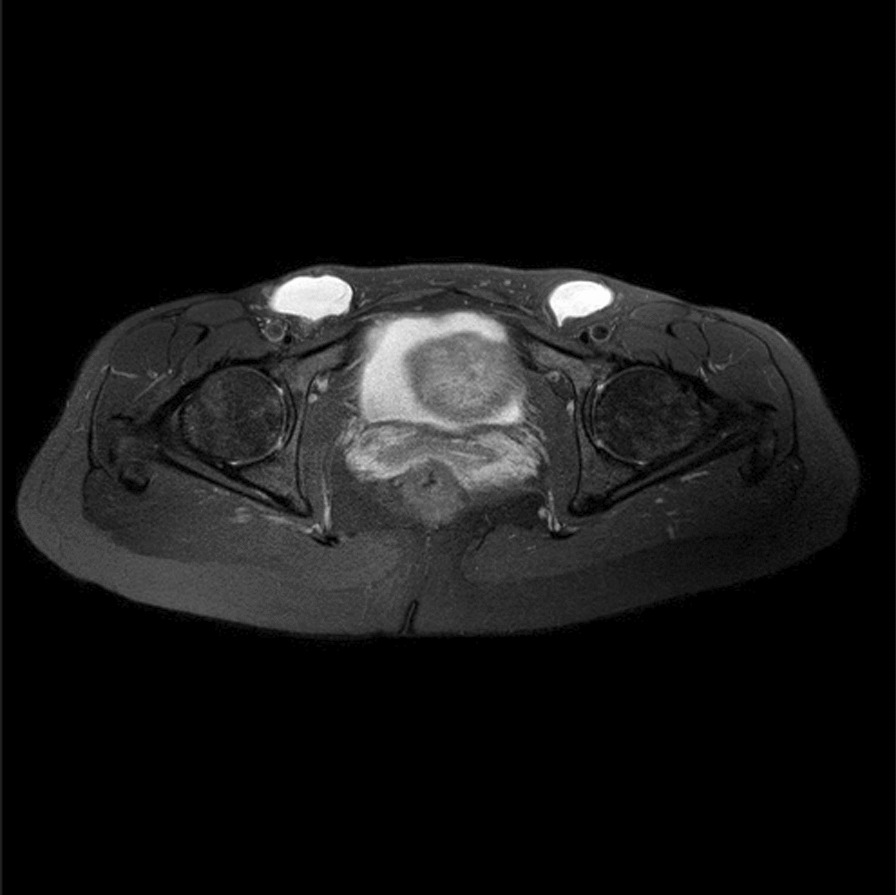
Preoperative MRI, T_2_ weighted, showing bilateral cystic protrusions of the groin

Physical examination found bilateral swelling of the inguinal canal without tenderness. The swellings did not disappear upon manipulation. A magnetic resonance imaging (MRI) scan of the hip and lower abdomen was performed, although ultrasound normally would be first choice image diagnostics. The MRI revealed cystic protrusions located subcutaneously in both groin areas. The cystic processes measured 83 × 37 × 48 mm on the right side and 67 × 30 × 30 mm on the left side, without communicating with other anatomical structures. The overall MRI concluded bilateral hydroceles of the canal of Nuck (Fig. 1).

Under general anesthesia, a bilateral excision of the cystic mass was performed. In both sides a groin exploration was performed, and the cysts were freed from their surroundings.

**Fig. 2:**
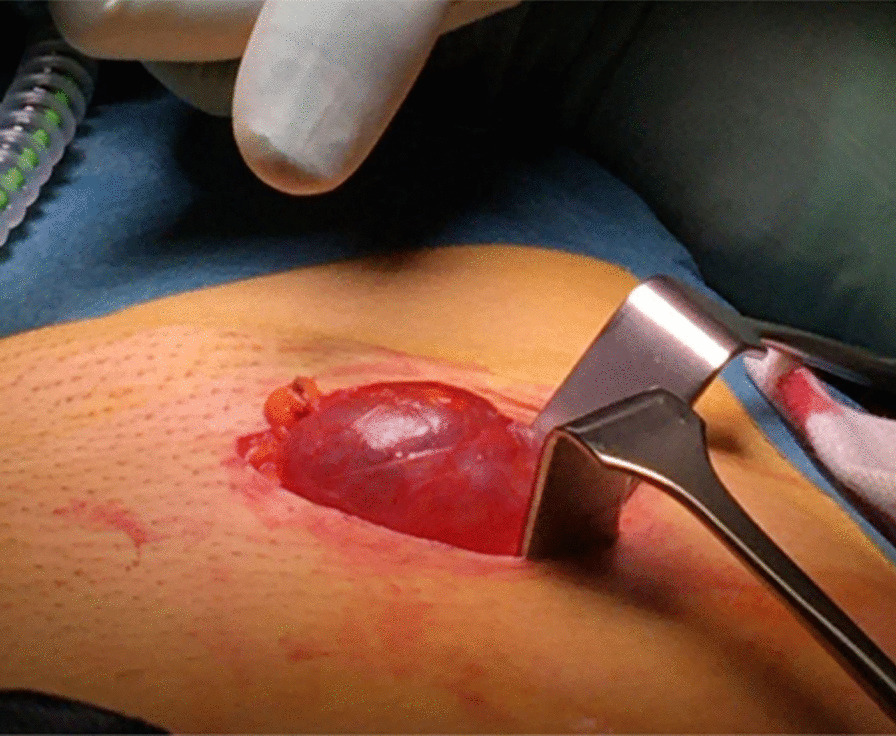
Right side groin exploration of a cystic process, revealing a hydrocele

**Fig. 3:**
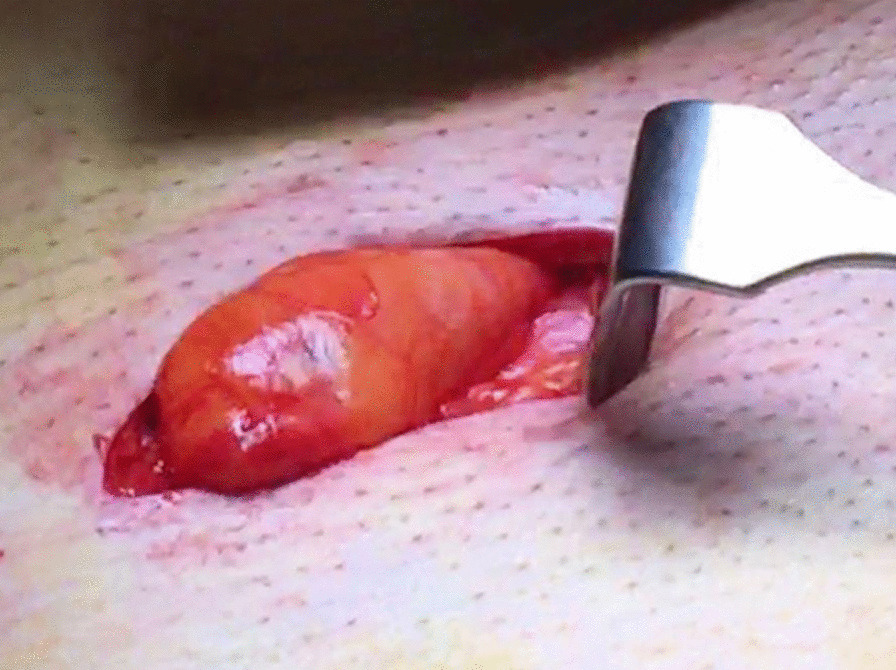
Left side groin exploration of a cystic process, revealing a hernia sac

Intraoperative findings included a hydrocele on the right side, measuring 120 × 40 × 30 mm, without communication with the peritoneal cavity. The hydrocele was punctured, producing clear water-like fluid, and excised according to its origin (Fig. 2). On the left side, a cystic process was detected, and throughout dissection a small intraabdominal communication was identified, resembling a medial groin hernia with a defect smaller than the diameter of the surgeon’s finger, assessed as a M1 hernia [4, 5]. After ensuring intraperitoneal relations, the cystic process was identified as a hernia and excised (Fig. 3). After discovery of a hernia, a diagnostic laparoscopy was performed to identify relation to femoral channel and assess potential mesh repair. Laparoscopy showed a left-sided medial inguinal hernia, with a defect measuring a few milliliters not requiring a mesh repair, and a right-sided impression of the described hydrocele, with no communications to the intraperitoneal space.

Subsequent histopathological examinations found on the right side a cyst wall with chronic inflammation, hemosiderin-laden macrophages, fibrosis and small tubular structures lined by a single layer of epithelial cells without atypia. The small tubular structures in the cyst wall showed positive calretinin staining confirming a mesothelial origin, (mesothelial entrapment). Histopathological examinations found on the left side flattened soft tissue with minor chronic inflammation lined with mesothelium without atypia.

The patient was discharged shortly after surgery. After follow-up 1 month and 3 months after surgery, the patient was well without signs of postoperative complications or hernia recurrence. The patient’s written consent was received and data were anonymized due to guidelines from the ethical committee.

## Discussion

In women, the prevalence of inguinal hernia is 3–5.8% [6], whereas groin hernia with onset during pregnancy has a prevalence of 0.12% [2]. Even though a female groin hernia has a risk of emergency procedures of 14.5% [7], such risk is not shown in groin hernias with onset during pregnancy [2]. Oma *et al*. analyzed groin hernias during pregnancy, and did not find any acute complications of hernia during pregnancy. A spontaneous remission was observed in 10 of 25 patients. In conclusion, a watchful waiting strategy was recommended [2].

Due to relatively high complication rates, the watchful waiting strategy is not recommended in groin hernias in women, with the exception of hernias debuting during pregnancy [7].

Hydroceles of the canal of Nuck are a rare condition, only previously described as case reports [1, 5]. These hydroceles, also defined as hydroceles in female patients, are often defined as cystic processes, which accompany the round ligaments through the inguinal canal [7]. On the basis of the reported cases, the mean age at which the condition is discovered is 35 years [1]. Hydroceles of the canal of Nuck can be classified into three types depending on the relation to its surroundings. Type I is described as not having any communication with the peritoneal cavity, type II communicates with the peritoneal cavity, and type III describes an hour-glass-shaped hydrocele, located partly in the peritoneal cavity [7]. Due to the rarity of the condition, the prevalence is unknown. Furthermore, no guidelines or recommendations in relation to diagnostics and choice of treatment exist.

In this case, preoperative MRI findings concluded bilateral type I hydrocele of the canal of Nuck. However, the operative findings were different, as only the right side was a type I hydrocele of the canal of Nuck, while the left side represented a M1 hernia. An abdominal bulge, not able to be repaired, was further diagnosed with ultrasound. When a cystic process has been identified, both hydrocele and inguinal hernia must be considered. Such diagnostic challenges can be supplemented with MRI or diagnostic laparoscopy to detect intraperitoneal communication. A confirmed inguinal hernia should be treated with reparation repair. If no mesh was implanted as recommended, the rationality should be argued. In this case report no mesh was implanted due to the very small defect, suitable for repair with suture only. The assessment to suture strategy were based on hernia size and medial type hernia, whereas it had not been possible in case of femoral hernia.

Hydroceles in the canal of Nuck have not been described during pregnancy until now. In Oma *et al*., patients were included from medical journals, including “bulge” or “hernia,” whereas some differential diagnosis (including cysts) may have been overlooked, and further classification was not made [2]. In our report, the groin bulge debuted during pregnancy, in line with Oma *et al*.’s study [2]. Our findings of hydrocele and hernia indicate that a “bulge” can be of other origins than a hernia.

It is recommended that a groin bulge debuting during pregnancy is treated by watchful waiting [2]. Such recommendations had been followed in the case described in this paper.

A hydrocele of the canal of Nuck is considered a safe condition, with no reports of complications [1]. Considering hydrocele as a condition with its debut during pregnancy, a “watchful waiting” strategy for inguinal bulges related to pregnancy could be of great clinical relevance. This report thus leaves the question of whether hydrocele in the canal of Nuck can be related to pregnancy. This question is difficult to answer due to the low prevalence of the condition.

In this case, a hydrocele and a hernia sac were morphologically identical in terms of preoperative appearance and development. Given the morphological correlation, it was surprising to find different operative findings confirmed by histopathological examination.

Our findings suggest a possible connection between the occurrence of hydroceles and hernia sacs. Such a connection has not yet been described in the literature.

## Conclusion

This is the first ever report of the synchronic development of two morphologically identical cystic processes, with one being a hydrocele and the other a hernia sac. In addition, the hydrocele developed during pregnancy, making this case even more unique.

## Data Availability

Not applicable.

## References

[1] Prodromidou A, Paspala A, Schizas D (2020). Cyst of the canal of Nuck in adult females: a case report and systematic review. Biomed Rep.

[2] Oma E, Bay-Nielsen M, Jensen KK (2017). Primary ventral or groin hernia in pregnancy: a cohort study of 20,714 women. Hernia.

[3] Köckerling F, Simons MP (2018). Current concepts of inguinal hernia repair. Visc Med.

[4] Nilsson H, Holmberg H, Nordin P (2018). Groin hernia repair in women—a nationwide register study. Am J Surg.

[5] Miserez M, Alexandre JH, Campanelli G (2007). The European hernia society groin hernia classification: simple and easy to remember. Hernia.

[6] Köckerling F, Koch A, Lorenz R (2019). Groin hernias in women—a review of the literature. Front Surg.

[7] Counseller VS, Black BM (1941). Hydrocele of the canal of Nuck: report of seventeen cases. Ann Surg.

